# Addition of ruxolitinib to standard graft-versus-host disease prophylaxis for allogeneic stem cell transplantation in aplastic anemia patients

**DOI:** 10.1038/s41409-024-02266-7

**Published:** 2024-04-05

**Authors:** Xiaoyu Zhang, Xiaoli Zhao, Shulian Chen, Mengze Hao, Lining Zhang, Ming Gong, Yuanyuan Shi, Jialin Wei, Ping Zhang, Sizhou Feng, Yi He, Erlie Jiang, Mingzhe Han

**Affiliations:** 1 State Key Laboratory of Experimental Hematology, National Clinical Research Center for Blood Diseases, Haihe Laboratory of Cell Ecosystem, Institute of Hematology & Blood Diseases Hospital, Chinese Academy of Medical Sciences & Peking Union Medical College, Tianjin, 300060 China; 2 Tianjin Institutes of Health Science, Tianjin, 301600 China; 3 Translational Science and Therapeutics Division, Fred Hutchinson Cancer Center, Seattle, WA 98109 USA

**Keywords:** Anaemia, Graft-versus-host disease

## Abstract

Allogeneic hematopoietic stem cell transplantation (allo-HSCT) offers rapid hematopoietic and immune reconstitution for aplastic anemia (AA). As a non-malignant disorder, attenuation of GVHD remains a clinical priority in AA patients. Our study sought to investigate the safety and efficacy of the prophylactic use of ruxolitinib in allogeneic HSCT. A total of 35 AA patients were retrospectively consecutively treated with allo-HSCT whereby ruxolitinib was added to the standard GVHD prophylaxis regimen (rux group). The addition of peri-transplant ruxolitinib did not impact the engraftment and graft function, while better recovery of CD4+ Tregs in the rux group was observed. Interestingly, the rux group demonstrated significantly lower incidence of bacterial/fungal infections (17.14% vs 45.71%). Compared to the control group, the rux group exhibited significantly lower incidence of moderate to severe aGVHD (17.1% vs 48.6%) with a trend toward lower severe aGVHD (8.6% vs 20%) and cGVHD (26.2 vs 38.3). The rux group also demonstrated a trend toward higher GVHD and failure-free survival (GFFS: 85.7% vs 68.6%) and lower TRM (2.9% vs 14.3%). Addition of ruxolitinib to standard GVHD prophylaxis regimen, thus, represents a safe and highly efficient method for the attenuation of GVHD with better outcome of allo-HSCT.

## Introduction

Aplastic anemia (AA) is a non-malignant hematological disorder characterized with hematopoietic failure and the severe cases (SAA) is often life-threatening. Allogeneic hematopoietic stem cell transplantation (allo-HSCT) and immunosuppressive therapy (IST) are the major curative therapeutic options for SAA or moderate AA. Compared with IST, allo-HSCT has the advantage of more rapid hematopoietic reconstitution with a lower risk of relapse and clonal evolution [1]. Furthermore, the development of HLA haplo-identical donor (HID) transplantation has significantly reduced the restrictions of donor selection [2]. Hence, allo-HSCT has become the first-line choice for AA, especially in younger patients [3, 4]. However, the high incidence of graft-versus-host disease (GVHD) and associated complications, especially in HID-HSCT, remains a major limitation for long-term survival and quality of life.

Ruxolitinib is a selective inhibitor of JAK1/2 signaling pathway and has demonstrate high efficacy in treating primary myelofibrosis [5, 6]. In the past decade, ruxolitinib is emerging as a potent modulator of GVHD [7] as the JAK/STAT pathway plays a crucial role in the initiation and progression of GVHD [8, 9]. Mechanistically, ruxolitinib reduces the release of pro-inflammatory cytokines and inhibits the proliferation of allogeneic T cells during GVHD. Further studies demonstrate attenuation of GVHD while preserving the graft-versus-tumor (GVT) effect following JAK1/2 inhibition [10, 11]. Ruxolitinib is also associated with the inhibition of interferon-γ receptor and interleukin 6 receptor signaling, leading to the increase of regulatory T-cells (Tregs) in the intestine [7]. Elegant clinical studies with ruxolitinib have proved high efficacy in treating steroid-refractory GVHD with tolerable side effects [12–14]. Hence, the US Food and Drug Administration has recently approved the use of ruxolitinib for chronic GVHD in adult and pediatric patients aged 12 years and older, who have not responded to one or two lines of systemic therapy.

Despite the efficacy in treating chronic GVHD, it remains unknown for ruxolitinib in the prophylaxis of acute GVHD. Only few studies have reported the prophylactic application of ruxolitinib after allogeneic HSCT, primarily in patients with myelofibrosis (MF) [15]. The majority of GVHD prophylaxis regimens focus on the inhibition of alloreactive T cells which is associated with the compromise of the GVT effect leading to a higher risk of relapse of underlying malignancies [16]. As a non-malignant disease, AA demonstrates the minimum risk of disease relapse following allo-HSCT thus representing an optimal candidate for active GVHD prophylaxis. Therefore, we conducted this pilot study to investigate the efficacy and safety of ruxolitinib in GVHD prophylaxis in AA patients which comprised of predominantly adults with a few pediatric patients. Here we show addition of ruxolitinib to a standard GVHD prophylaxis regimen has resulted in a significantly lower incidence of acute GVHD, faster immune reconstitution, and attenuated infectious complications.

## Methods

### Patient population

This retrospective, single-center case series included 35 consecutive AA patients who underwent allo-HSCT and received ruxolitinib as additional GVHD prophylaxis between August 2021 and January 2023 at the Stem Cell Transplantation Center, Institute of Hematology and Blood Diseases Hospital, Chinese Academy of Medical Sciences. This observational study was part of a larger study, the “NICHE‐BMT” project (a retrospective analysis of IHCAMS recipients of bone marrow transplantation), which was approved by the IHCAMS Clinical Research Academic Committee and the IHCAMS Ethics Committee on February 7, 2021 (IIT2021011-EC-1). The historical control cohort consisted of consecutively treated AA patients (*n* = 76) who received allo-HSCT in our center from January 2019 to June 2021. All patients in the control cohort didn’t receive peri-transplant ruxolitinib otherwise followed the same treatment protocol. Congenital bone marrow failure was excluded. Fanconi anemia was excluded based on the chromosome breakage and gene test. The presence of PNH clone was confirmed with flow cytometry. PNH testing was performed by FLAER-based assay according to PNH Consensus Guidelines [17, 18]. FLAER/CD33/CD15/CD45 and FLAER/CD59 panels were used for white blood cell and red blood cell testing, respectively.

For safety and response evaluation, all data were collected from clinical records. Informed consent was obtained from all participants before ruxolitinib treatment and data collection. This study was approved by the Ethics Committee of the Institute of Hematology and Blood Diseases Hospital and all patients or guardians provided informed written consent in accordance with the Declaration of Helsinki. This study followed the reporting guidelines for case series.

### Conditioning regimens and GVHD prophylaxis

HSCT recipients were conditioned as previously described including FAC or BFAC regimens [19]. The FAC conditioning regimen was comprised of fludarabine (30 mg/m^2^/d, days −5 to −1), cyclophosphamide (30 mg/kg/d or 37.5 mg/kg/d, days −5 to −2), and rabbit anti-thymocyte globulin (rATG 2.5 mg/kg/d, days −5 to −1) or porcine anti-lymphocyte globulin (pALG 20 mg/kg/d, days −5 to −1). The BFAC conditioning regimen included busulfan (3.2 mg/kg/d, days −7 to −6) on top of FAC. Details of GVHD prophylaxis and supportive care are consistent with the previous experience [19]. All recipients received cyclosporine A or tacrolimus, short-term methotrexate as GVHD prophylaxis. HID-HSCT recipients received mycophenolate mofetil consistent with previous experience, compared with HLA-matched sibling donor-HSCT (MSD-HSCT). Ruxolitinib was initiated at the start of the conditioning regimen and continued until 3 months post-transplantation with a dose of 5 mg twice daily. The dose of ruxolitinib was reduced from 10 mg/day to 5 mg/day (5 mg once daily) in case of severe adverse events according to the Common Terminology Criteria for Adverse Events version 4 (CTCAE v4). All patients received granulocyte colony-stimulating factor (G-CSF, 5 μg/kg/day, subcutaneously) from day +7 post-HSCT until neutrophil recovery. The thrombopoietin (TPO) was routinely applied at at a daily dose of 15,000 U from day +1 for 1 month according to the Chinese guidelines and reported clinical trial results [20–22]. Routine anti-fungal prophylaxis was started on the day of transplantation. Patients were given oral posaconazole (300 mg once daily) or oral voriconazole (200 mg twice daily) for the duration of neutropenia (neutrophil count ≤500) [23] and up to 100 days which were extended in the presence of risk factors of invasive fungal infections. Patients received regular CMV and EBV PCR testing twice a week until 100-day post-HSCT, then once a week up to day 200 post-HSCT. Frequency of additional viral screening after day +200 is based on the presence of risk factors including occurrence of GVHD, use of steroids, and delay of immune reconstitution. CMV PCR threshold for pre-emptive treatment was defined as 1000 copies/ml. EBV PCR threshold for pre-emptive treatment was defined as 500 copies/ml.

### Outcome assessment

The primary objective of this study is to evaluate the cumulative incidence of grades II–IV acute GVHD (moderate to severe aGVHD). The secondary objectives include neutrophil and platelet (PLT) engraftment, donor/recipient chimerism, immune reconstitution, infectious complications, incidence of chronic GVHD (cGVHD), and survival. The grade of aGVHD and cGVHD was assessed following standard international criteria [24, 25]. Neutrophil recovery was defined as having 3 consecutive days with an absolute neutrophil count (ANC) greater than 0.5 × 10^9^/L. PLT engraftment was defined as the first day with a sustained PLT count above 20 × 10^9^/L without any PLT transfusions in the preceding 7 days. Primary graft failure (GF) was defined as the failure of myeloid engraftment until day +28. Secondary poor graft function (sPGF) was defined as recurrent pancytopenia with obviously hypocellular BM after achieving a documented engraftment in the presence of full donor chimerism, and absence of severe GvHD, active infection, and drug toxicity [26–28]. Failure-free survival (FFS) refers to survival with complete response whereas death, GF, and relapse are considered treatment failures. GFFS (GVHD-free, FFS) is defined as survival without the occurrence of grades III–IV acute GVHD, extensive chronic GVHD or treatment failures as mentioned above. Transplantation-related mortality (TRM) was defined as death without GF.

### Statistical analysis

Patient characteristics were compared using chi-square or Fisher’s exact tests for binary variables and Mann–Whitney *U*-test for continual variables. The probabilities of OS, FFS, and GFFS are calculated using the Kaplan–Meier method and the difference between cohorts is estimated with the log-rank method (R version 4.2.3, http://www.r-project.org). The probabilities of neutrophil and PLT engraftment, TRM, and aGVHD and cGVHD incidence are calculated using the cumulative incidence method (R version 4.2.3, http://www.r-project.org). Two differentially distributed variables i.e., conditioning regimens (regimen w/o Bu) and ATG types (rATG or pALG) were Included in the propensity score matching (PSM) analysis to identify patients from historical cohort to match the ruxolitinib cohort. A 1:1 nearest neighbor matching with a caliper width of 0.2 [29] with significance level at *P* < 0.05 were adopted.

## Results

### Patient characteristics

A total of 35 patients (MSD-HSCT *n* = 16, HID-HSCT *n* = 19) were consecutively enrolled for peri-transplant ruxolitinib prophylaxis from August 2021 to March 2023 with basic demographic characteristics listed in Table 1. The median age of the cohort (hereafter refer to rux group) was 27 (range 12, 57) including five patients that are older than 40 and four pediatric patients (<18). Seven patients (20%) were diagnosed with moderate AA. Among the very severe AA (VSAA) or SAA patients (*n* = 28), three patients failed prior treatment with ATG-based IST. The majority of the patients (32/36, 88.9%) were fully active before treatment with Karnofsky performance status (KPS) scores over 80. Nine patients had a history of infection before HSCT and all achieved PR or CR at transplantation. BFAC was the dominant conditioning regimen (29/35, 82.9%). All patients received peripheral blood stem cells (PBSC) whereby two patients received cord blood at +4d post-HSCT due to low quality of stem cell graft. The median duration of ruxolitinib administration was 3 months (range: 3–6 months). Dose reduction was required in only one patient who developed thrombocytopenia.

**Table 1:** Basic characteristics of patients receiving ruxolitinib for GVHD prevention.

Characteristic	Ruxolitinib prophylaxis group, *N* = 35(%)
Age, median (range), year	27 (17.36)
Older than 40 y	5 (14.29)
Gender male/female	16 (45.71)/19 (54.29)
Diagnosis	
SAA/VSAA	28 (80)
Moderate AA	7 (20)
HCT-CI (0~1)	32/(91.43)
KPS score (≥80)	32/(91.43)
CCB at diagnosis, median (range)	
WBC (×10^9^/L)	1.7 (1.4, 2.4)
Hb (g/L)	62 (51, 77)
PLT (×10^9^/L)	9 (4, 16)
ANC (×10^9^/L)	0.46 (0.2, 0.8)
Bacterial/fungal infections at HSCT	9 (25.71)
Bloodstream	2/9
Pneumonia	5/9
Skin and soft tissue	2/9
FUO	2/9
The interval from diagnosis to HSCT, median (range), day	90 (45, 730)
Treatment before HSCT	
ATG	3 (8.57)
TPO agonists	6 (17.14)
CSA or FK506 as IST	28 (80)
PNH clone	3 (8.57)
Donor type	
MSD	16 (45.71)
HID	19 (54.29)
ABO compatibility/incompatibility	20 (57.14)/15 (42.86)
Median MNC graft infused: 10^8^/kg of recipient	10.41
Median CD34 stem cells graft infused: 10^6^/kg of recipient	4.55
Conditioning regimen	
Cy + Flu + ATG	5 (14.29)
Bu + Cy + Flu + ATG	29 (82.86)
TBI + Bu + Cy + Flu + ATG	1 (2.85)
rATG/pALG	20 (57.14)/15 (42.86)
Cord blood stem cell infusion	2 (5.71)

Categorical variables are presented as number(percentiles); continuous variables are presented as median (interquartile range) unless otherwise stated.

*SAA/VSAA* severe/very severe aplastic anemia, *HCT-CI* hematopoietic cell transplant-comorbidity index, *KPS score* Karnofsky performance status, *CCB* cell count of blood, *WBC* white blood cell, *Hb* hemoglobin, *PLT* platelet, *ANC* absolute neutrophil count, *FUO* fever of unknown origin disease, *TPO* thrombopoietin, *CSA* cyclosporin, *FK506* tacrolimus, *MSD* matched sibling donor, *HID* haplo-identical donor.

There are 76 AA patients in the historical control cohort who underwent HSCT consecutively in our center from January 2019 to June 2021. The historical control group and ruxolitinib group are comparable in age, gender, disease diagnosis, KPS score, history of infections, and donor type. Unfortunately, the conditioning regimen is significantly different between the historical control group and the ruxolitinib group (Supplementary Table 1) whereby higher proportion of the historical cohort used ALG (64.47% vs 42.86) and FAC (39.47% vs 14.29%) as conditioning regimen. We thus performed propensity score matching [29] to minimize the effects of confounding factors resulting in 35 patients in the historical-matched-control group (hereafter refer to control group). The matched patients were not significantly different in terms of baseline characteristics compared to ruxolitinib group, which are shown in Table 2.

**Table 2:** Baseline characteristics between history-matched-control and ruxolitinib group.

Variable	Historic control group no. (%) (*N* = 35)	Ruxolitinib group no. (%) (*N* = 35)	*p*
Gender			0.473
Male	20 (57.14)	16 (45.71)	
Female	15 (42.86)	19 (54.29)	
Age, median (range) (years)	27 (17.36)	25 (17.29)	0.72
Age > 40 (years)	6 (17.14)	5 (14.29)	1.000
Diagnosis			
VSAA/SAA	30 (85.71, 17/13)	28 (80, 15/13)	0.752
Moderate AA	5 (14.29)	7 (20)	
ATG before HSCT	6 (17.14)	3 (8.57)	0.477
PNH clone	7 (20)	3 (8.57)	0.306
HSCT type			0.809
MSD-HSCT	14 (40)	16 (45.71)	
HID-HSCT	21 (60)	19 (54.29)	
Infection before HSCT	8 (22.86)	9 (25.71)	1.000
ATG type			
rATG	20 (57.14)	20 (57.14)	1.000
pALG	15 (42.86)	15 (42.86)	
Conditioning regimen			
FAC	5 (14.29)	5 (14.29)	1.000
BFAC	30 (85.71)	30 (85.71)	

Categorical variables are presented as number(percentiles); continuous variables are presented as median (interquartile range) unless otherwise stated.

*SAA/VSAA* severe/very severe aplastic anemia, *PNH* paroxysmal nocturnal hemoglobinuria, *MSD* matched sibling donor, *HID* haplo-identical donor, *rATG* rabbit anti-thymocyte globulin, *pALG* porcine anti-lymphocyte globulin.

### Engraftment

**Fig. 1:**
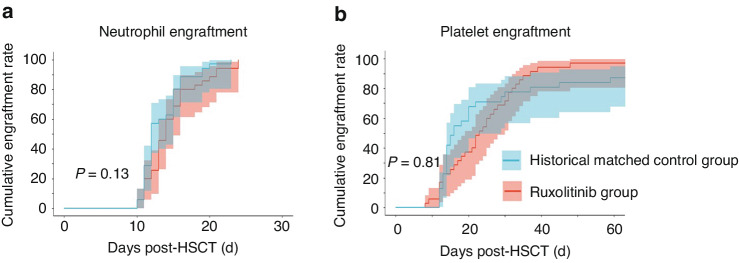
Hematopoietic engraftment after allogeneic HSCT. Cumulative incidence of engraftment for **a** neutrophil (count > 0.5 × 10^9^/L) and **b** platelet (count > 20 × 10^9^/L) in the rux and the control groups (*n* = 35 per group).

Within 28 days post-HSCT, all patients in the rux cohort achieved myeloid engraftment at a median time of 14 (10–24) days whereas 24 patients (68.6%) had PLT engraftment with a median time of 23 (8–112) days. No primary engraftment failure was recorded. Two patients experienced sPGF, demonstrating as cytopenia with full donor chimerism which was rescued by CD34+ stem cell transfusion. All patients achieved sustained full donor chimerism at 3 months post-HSCT. We further compared these parameters to that of the control group. The cumulative incidence of neutrophil engraftment was 100% in both groups by day +28 (Fig. 1a). In contrast, PLT engraftment tends to be higher in the rux group at day +60 which were 97.1% (95% CI 80.3–99.6%) in the rux group and 83.9% (95% CI 64–92.8%) in the control group respectively (*P* = 0.81) (Fig. 1b). The rux and control groups had two and three cases of secondary PGF respectively (Table 3) with three cases in the control group being secondary to GVHD treatment.

**Table 3:** Characteristics of HSCT outcomes between history-matched-control and ruxolitinib group.

Variable	Historic control group no. (%) (*N* = 35)	Ruxolitinib group no. (%) (*N* = 35)	*p*
28-day neutrophil engraftment rate, median (extreme range)	35 (100), 12 (10–23)	35 (100), 14 (10–24)	0.145
28-day platelet engraftment rate, median (extreme range)	22 (62.85), 16 (12–1501)	24 (68.57), 23 (8–112)	0.377
Poor graft function	5 (14.29)	2 (5.71)	0.428
Bacterial/fungal Infection	16 (45.71)	6 (17.14)	0.019^a^
CMV reactivation	16 (45.71)	16 (45.71)	1.000
II~IV aGVHD	17 (48.57)	6 (17.14)	0.010^a^
III~IV aGVHD	7 (20)	3 (8.57)	0.306
Skin involved aGVHD	8/17	2/6	
Liver involved aGVHD	4/17	1/6	
Intestine involved aGVHD	9/17	6/6	
II~IV aGVHD onset timepoint, median (range)	30 (13–90)	60 (26~90)	0.122
cGVHD	13 (37.14)	6 (17.14)	0.054
Extensive cGVHD	2/13	1/6	
The proportion of donor chimerism at 1 m post-HSCT, median (extreme range)	99.72% (91.74–99.98%) (*n*^b^ = 30)	99.15% (96.5–100%) (*n*^b^ = 30)	0.925
The proportion of donor chimerism at 1 y post-HSCT, median (extreme range)	99.66% (95.45–99.97%) (*n*^b^ = 30)	99.15% (98.8–100%) (*n*^b^= 30)	0.963

Categorical variables are presented as number(percentiles); continuous variables are presented as median (interquartile range) unless otherwise stated.

*CMV* cytomegalovirus, *aGVHD* acute graft-versus-host disease, *cGVHD* chronic graft-versus-host disease.

^a^Statistical significance for the factors.

^b^Actual number of patients evaluable for analysis.

### Immune reconstitution and infectious complications

**Fig. 2:**
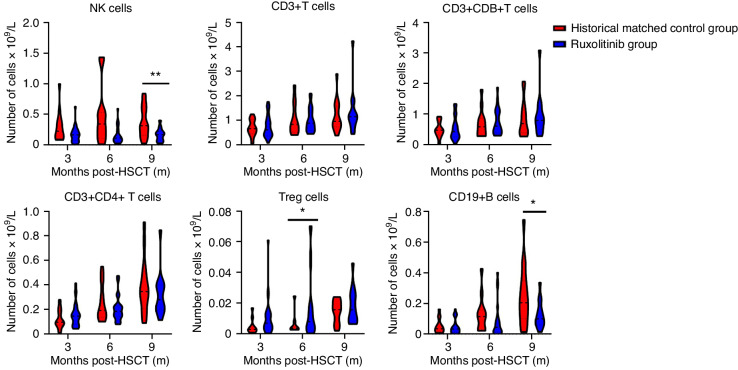
Immune reconstitution following allogeneic HSCT. Numbers of major lymphocyte subsets in the peripheral blood over time. Data are presented as median ± interquartile range (*n* = 16 in the control and *n* = 28 in the rux groups respectively). *P value < 0.05.

Having established that ruxolitinib didn’t have negative impact on the engraftment, we further investigated the immune reconstitution by examining the recovery of major lymphocyte subsets in the peripheral blood. The rux group demonstrated rapid NK cell recovery within 3 months whereas the main lymphocyte subsets (T cells and CD19+ B cells) had progressive recovery over time (Supplementary Fig. 1). We further compared the absolute counts of NK cells, CD3+, CD3+CD8+, and CD3+CD4+ T cells, CD4+ Tregs, and CD19+ B cells to that of the control groups at 3, 6, and 9 months after HSCT. The rux and the control group demonstrated similar levels of recovery for CD3+, CD3+CD8+, and CD3+CD4+ T cells (Fig. 2). Of note, the rux group demonstrated more rapid recovery of CD4+ Tregs with a trend of higher counts at 3 months significantly higher counts at 6 months after HSCT (Fig. 2 and Supplementary Fig. 2). The increased Treg recovery is consistent with previous studies [30] and may contribute to superior control of GVHD. Unexpectedly, the rux group had delayed recovery of CD19+ B cells and NK cells (Fig. 2 and Supplementary Fig. 2) which warrants further investigation.

We next ask if ruxolitinib prophylaxis impact infectious complications after HSCT. In the rux group, we recorded seven cases of bacterial/fungus infections among which four had a history of infection prior to HSCT. One patient died of severe pancreatitis and Isoniazid associated multi-organ dysfunction secondary to mycobacterium tuberculosis (TB) septicemia. Intriguingly, the bacterial/fungal infection is significantly lower than that of the control group which is 17.1% and 45.7%, respectively (*P* = 0.02, Table 3). However, there is no difference for cytomegalovirus (CMV) reactivation (both groups are 45.7%) or Epstein-Barr virus (EBV) infections (Table 3). No other viral infections were documented. Hence, addition of ruxolitinib to GVHD prophylaxis does not have significantly negative impact on immune reconstitution.

### GVHD

**Fig. 3:**
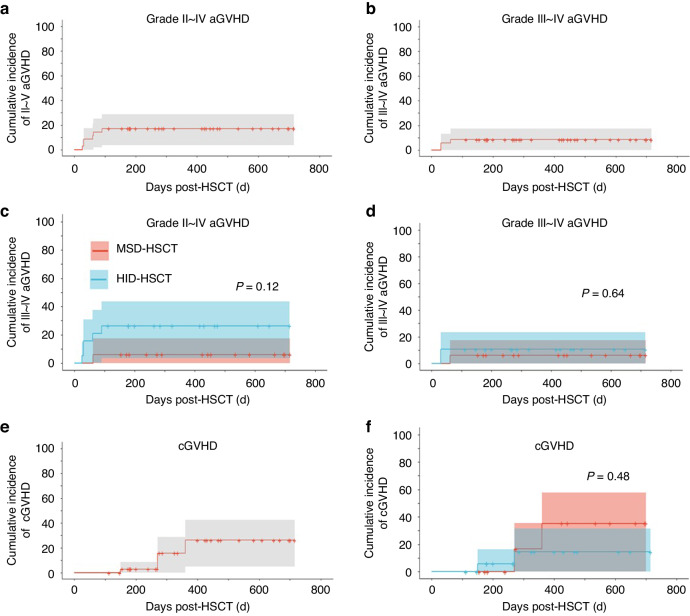
Incidence of GVHD after allogeneic HSCT in the rux cohort. Cumulative incidence of grades II–IV (**a**) and grades III–IV aGVHD (**b**) in the rux cohort (*n* = 35). Cumulative incidence of grades II–IV (**c**) and grades III–IV aGVHD (**d**) in recipients of MSD-HSCT (*n* = 16) vs HID-HSCT (*n* = 19). Cumulative incidence of cGVHD in the rux cohort (**e**) and that in the recipients of MSD-HSCT (*n* = 16) vs HID-HSCT (*n* = 19) (**f**). *P value < 0.05.

**Fig. 4:**
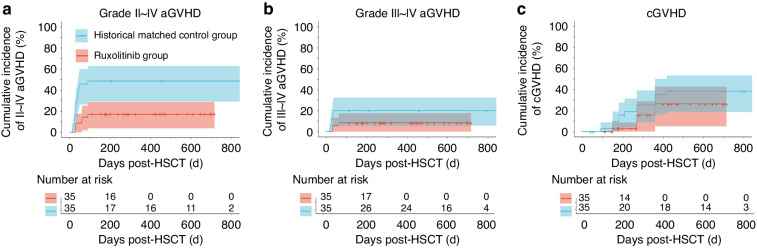
Incidence of GVHD after allogeneic HSCT in the rux and the control groups. Cumulative incidence of grades II–IV aGVHD (**a**), grades III–IV aGVHD (**b**), and cGVHD (**c**) in the rux and the control groups (*n* = 35 per group). *P value < 0.05.

The primary objective of the present study is to investigate the impact of peri-transplant ruxolitinib administration on the incidence of GVHD. The rux group demonstrated cumulative incidence of grades II–IV aGVHD and grades III–IV aGVHD at 17.1% (95% CI 3.7–28.7%) and 8.6% (95% CI 0–17.4%), respectively (Fig. 3a, b). The median time to onset for any grades aGVHD is 60 (26–90) days. In the MSD-HCST recipients, only one patient developed grade III aGVHD which predominantly affected the intestine. As expected, the HID-HSCT recipients demonstrated a higher incidence of aGVHD with cumulative incidence of grades II–IV aGVHD and III–IV aGVHD at 26.3% (95% CI 3.6–43.7%) and 10.5% (95% CI 0 to ~23.3%), respectively (Fig. 3c, d). Of note, all cases of aGVHD including grades III–IV aGVHD resolved following treatment with a median time of 37.5 (14–90) days and no recipients died of aGVHD or associated complications. Compared to the control group, the rux group exhibited significantly lower incidence of moderate to severe (grade II~IV: 17.1% vs 48.6%, *P* = 0.033) or severe (grade III–IV: 8.6% vs 20%, *P* = 0.16) aGVHD (Fig. 4a, b).

Following the encouraging results in aGVHD, we further analyzed the incidence of cGVHD in the two cohorts. The rux group had a cumulative incidence at 26.2% (95% CI 5.2–42.6%) with the majority of cases demonstrating mild to moderate cGVHD. Surprisingly, the HID-HSCT recipients demonstrated lower incidence as compared to MSD-HSCT recipients (14.4% vs 35.2%, *P* = 0.48, Fig. 3e, f). Only one patient developed severe cGVHD which was diagnosed as bronchiolitis obliterans syndrome at day +120. Similar to that of aGVHD, the rux group exhibited a trend toward lower incidence of cGVHD as compared to the control group (26.2% vs 38.3%, *P* = 0.24, Fig. 4c). Thus, our data suggest the efficacy of peri-transplant ruxolitinib in preventing acute GVHD.

### Transplant-related mortality and long-term outcome

**Fig. 5:**
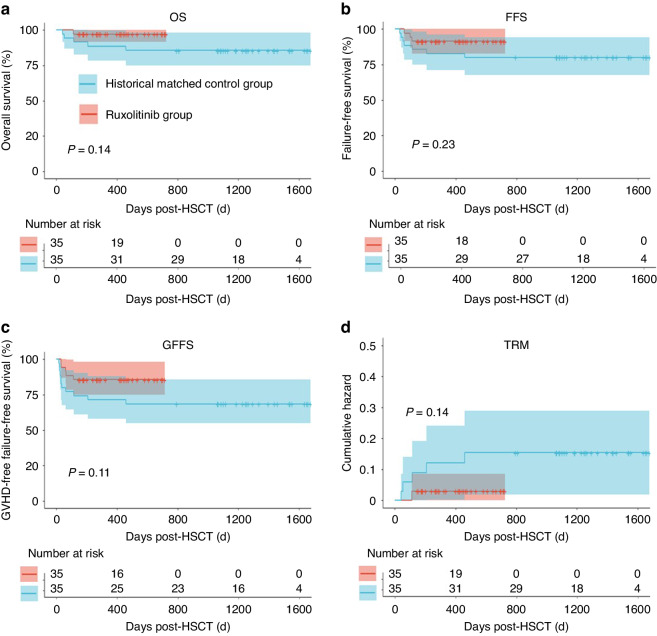
Survival analysis in the rux and the control groups. Overall Survival (**a**), Failure-free Survival (**b**), GVHD-free Failure-free Survival (**c**), and transplant-related mortality (**d**) in the rux and the control groups (*n* = 35 per group). *P value < 0.05.

At a median follow-up of 417 days (range, 112–725), only one patient in the rux group died of TB-associated complications on day +112 corresponding to transplantation-related mortality (TRM) of 2.9% (95% CI 0–8.2%). The cohort thus demonstrate 1-year OS, FFS, and GFFS at 97.1% (95% CI 91.8–100%), 91.4% (95% CI 82.6–100%), and 85.7% (95% CI 74.9–98.1%), respectively (Fig. 5). In the control group, GVHD (*n* = 3) and severe bacterial infections (*n* = 2) were the two major causes of death, contributing to TRM of 14.3% (95% CI 1.9–25.1%). Notably, the two patients who died of severe infection experienced prior sPGF. Despite lack of statistical significance, we noted more than 10% advantage of GVHD FFS in the rux group (85.7% vs 68.6%, *P* = 0.18). This data suggest the potential reduction of GVHD and infectious complications following addition of ruxolitinib to standard GVHD prophylaxis regimen.

## Discussion

Allogeneic HSCT represents therapeutic option for AA patients offering rapid hematopoietic reconstitution and sustained wellness which demonstrate superior advantage to conventional IST in younger patients [19]. As a non-malignant hematological disease, the primary goal of allogeneic HSCT for AA patients is to achieve hematopoietic and immune reconstitution with minimum GVHD and associated complications [31]. The mainstream of active GVHD prophylaxis includes PTCy- and G-CSF/ATG-based protocols [32, 33]. While PT/Cy-based protocol has the advantage of lower II–III aGVHD, it increases the risk of GF [34]. In contrast, the G-CSF/ATG-based protocol, with infused grafts consisting of G-CSF-primed BM and PBSCs, has a very low risk of GF with moderate to severe aGVHD at a range of 20–40% [35, 36]. G-CSF/ATG-based protocols remain the mainstay in China [37] where over 12,700 cases of allogeneic HSCT were conducted in 2021 [38]. Though BM is superior to PBSC in reducing GVHD, PBSC allografts result in a lower risk of GF and early neutrophil engraftment, benefiting patients with severe infections [39]. Moreover, BM grafts are not immediately available in a large fraction of HSCT especially unrelated donor HSCT which is rapidly increasing due to decreasing family size. Therefore, optimization of the standard GVHD prophylaxis regimens in PBSC-only HSCT represents an unmet clinical need and is also an ongoing effort. In the past decade, a number of methods have proved efficacy in the optimization of current GVHD prophylaxis regimens including the addition of tocilizumab [40] and vedolizumab [41] et al. As a selective JAK1/2 signaling inhibitor, ruxolitinib has been proven effective in the treatment of steroid-refractory GVHD [12, 13]. In this study, we incorporated peri-transplant ruxolitinib into the standard G-CSF/ATG-based GVHD prophylaxis regimen in AA patients who underwent MSD- or HID-HSCT receiving only PBSC grafts.

Of note, ruxolitinib was initiated at the beginning of the conditioning regimen which continued until 3 months after transplantation when the recipients are in high-risk of aGVHD. GF is a primary concern for peri-transplant ruxolitinib; however, our study demonstrated that it does not have negative impact on the hematopoietic and immune reconstitution or sPGF. This is consistent with a prior murine study whereby ruxolitinib promotes the function of bone marrow mesenchymal stromal cells during aGVHD and enhances their ability to support donor-derived hematopoiesis [42]. Additionally, ruxolitinib significantly promoted the reconstitution of CD4+ Tregs. This aligns with previous researches showing that frequencies of IFNg-producing CD4+ T cells were reduced through STAT1–JAK1/2-mediated signaling whereas Tregs were increased in recipients of ruxolitinib treatment [7, 43]. Tregs play critical role in immune tolerance thus their accelerated recovery may have a protective role for GF in AA patients and also attenuates GVHD. In addition to these well recognized mechanisms, ruxolitinib also demonstrates immunomodulatory effect on T-cell activation for example through the inhibition of MHC-II expression of neutrophils [44] or suppression of dendritic cell differentiation and function [45].

There is no consensus as to the impact of peri-transplant ruxolitinib on the risk of infections. A number of studies suggested that ruxolitinib may increase the risk of infections especially CMV reactivation [46] whereas others did not confirm an impact on infection rates during the treatment or prevention of GVHD [47]. Strikingly, addition of ruxolitinib significantly reduced the incidence of bacterial/fungal infections in our study. This protection on infection may be a result of lower incidence of GVHD and associated immune suppressive therapy.

There are promising results for the prophylactic use of ruxolitinib in preclinical studies [7]. However, clinical evidence regarding the safety and efficacy of ruxolitinib in preventing GVHD remains limited [48, 49]. In the clinical investigation by Huang et al., ruxolitinib represents a promising replacement for calcineurin inhibitors-intolerance in GVHD prevention [50]. In another study by Kröger et al., peri-transplant application of ruxolitinib until stable engraftment in MF patients [15], led to low incidence of grade II–IV aGVHD (8% by day +100) without cases of NRM. Elena et al. investigated the addition of ruxolitinib to PT/Cy in MF patients [51] leading to an incidence of grade II–IV acute GVHD of 25% with grade III–IV GVHD at 15%. However, over 50% (11/20) of the recipients experienced poor graft function and required dosage reduction for ruxolitinib. Our study is the first to explore the peri-transplant use of ruxolitinib in addition to standard GVHD prophylaxis in AA patients. Compared to a historical-matched control cohort, addition of ruxolitinib is safe and well-tolerated without delaying engraftment or immune reconstitution. As expected, ruxolitinib significantly decreased the incidence of moderate to severe aGVHD leading to potential improvement in GFFS, delay of aGVHD onset and high response rate of severe aGVHD treatment. In all, the addition of ruxolitinib to standard GVHD prophylaxis regimen demonstrated high safety and indicated profound efficacy in reducing aGVHD. While the historical cohort demonstrated very high incidence of GVHD, addition of ruxolitinb has decreased this risk to a level comparable to that of the PT-Cy-based protocols [34]. The optimization of PBSC-based transplant protocols is particularly beneficial for recipients who do not have to access to bone marrow graft.

Deeper findings are limited by the retrospective nature and small cohort size of the current study. Hence, we have started a prospective study (NCT05914714) to further investigate the safety and efficacy of peri-transplant ruxolitinib in the prevention of GVHD in AA patients. More solid conclusions on the prophylactic use of ruxolitinib will be benefited from results of a number of large-scale, randomized clinical trials (for example NCT06008808 and NCT 03286530). In sum, addition of ruxolitinib to standard GVHD prophylaxis regimen represents a safe and highly efficient method to improve the outcome of allogeneic HSCT and deserves deeper investigations.

### Supplementary information

Supplementary Data

## Data Availability

All data generated or analyzed during this study are included in this published article.
