# Supplementary material for: Addition of ruxolitinib to standard graft-versus-host disease prophylaxis for allogeneic stem cell transplantation in aplastic anemia patients
Source: Bone Marrow Transplant. 2024 Apr 5;59(7):997–1005. doi: 10.1038/s41409-024-02266-7 (PMC11226399; doi:10.1038/s41409-024-02266-7)

**Supplementary Table 1. Baseline characteristics between historical-control and ruxolitinib group**

| Variable | Historical-control group  No. (%)  (N=76) | Ruxolitinib group  No. (%)  (N=35) | p |
| --- | --- | --- | --- |
| Gender |  |  | 0.306 |
| Male | 44 (57.89) | 16 (45.71) |  |
| Female | 32 (42.11) | 19 (54.29) |  |
| Age>40 | 11 (14.47) | 5 (14.29) | 1.000 |
| Diagnosis |  |  |  |
| VSAA/SAA | 68 (89.47) | 28 (80) | 0.232 |
| moderate AA | 8 (10.53) | 7 (20) |  |
| ATG before HSCT | 9 (11.84) | 3 (8.57) | 0.750 |
| PNH | 17 (22.37) | 3 (8.57) | 0.111 |
| HSCT type |  |  | 1.000 |
| MSD-HSCT | 35 (46.05) | 16 (45.71) |  |
| HID-HSCT | 41 (53.95) | 19 (54.29) |  |
| Infection before HSCT | 14 (18.42) | 9 (25.71) | 1.000 |
| ATG type |  |  |  |
| rATG | 27 (35.53) | 20 (57.14) | 0.040* |
| pALG | 49 (64.47) | 15 (42.86) |  |
| Conditioning regimen |  |  |  |
| FAC | 30 (39.47) | 5 (14.29) | 0.08* |
| BFAC | 46 (60.53) | 30 (85.71) |  |

Abbreviations: SAA/VSAA, severe/very severe aplastic anemia; PNH, paroxysmal nocturnal hemoglobinuria; MSD, matched sibling donor; HID, haplo-identical donor; rATG, rabbit anti-thymocyte globulin; pALG, porcine anti-lymphocyte globulin.

.

**Figure legends**

Supplementary Figure 1. Immune reconstitution following allogeneic HSCT in the ruxolitinib group. Numbers of major lymphocyte subsets in the peripheral blood over time. Data are presented as median +/- interquartile range (*n* = 28). * P value < 0.05.

Supplementary Figure 2. Immune reconstitution following allogeneic HSCT. Proportions of major lymphocyte subsets in the peripheral blood over time. Data are presented as median +/- interquartile range (*n* = 16 in the control and *n* = 28 in the rux groups respectively). * P value < 0.05.

Supplementary Figure 1


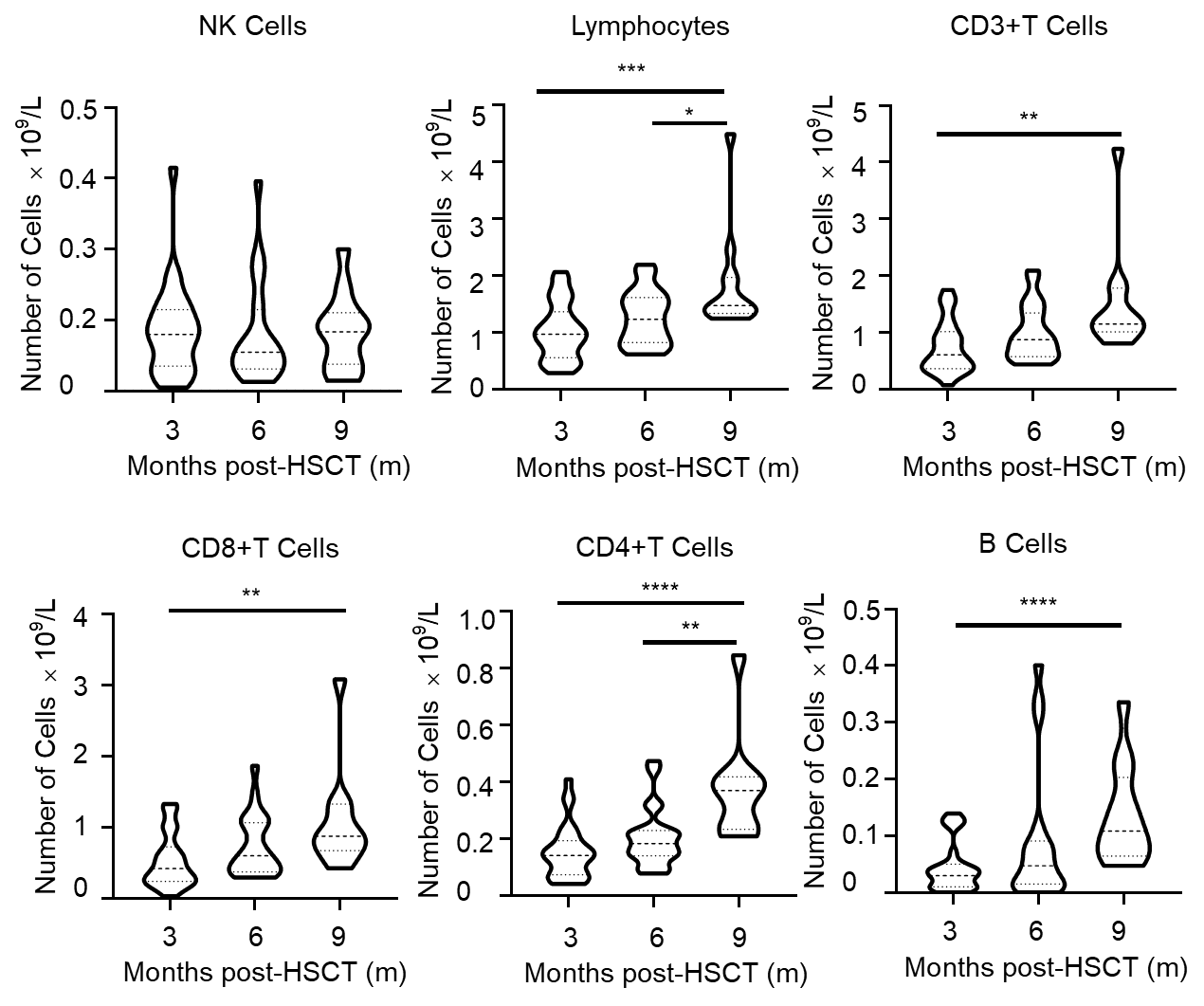


Supplementary Figure 2


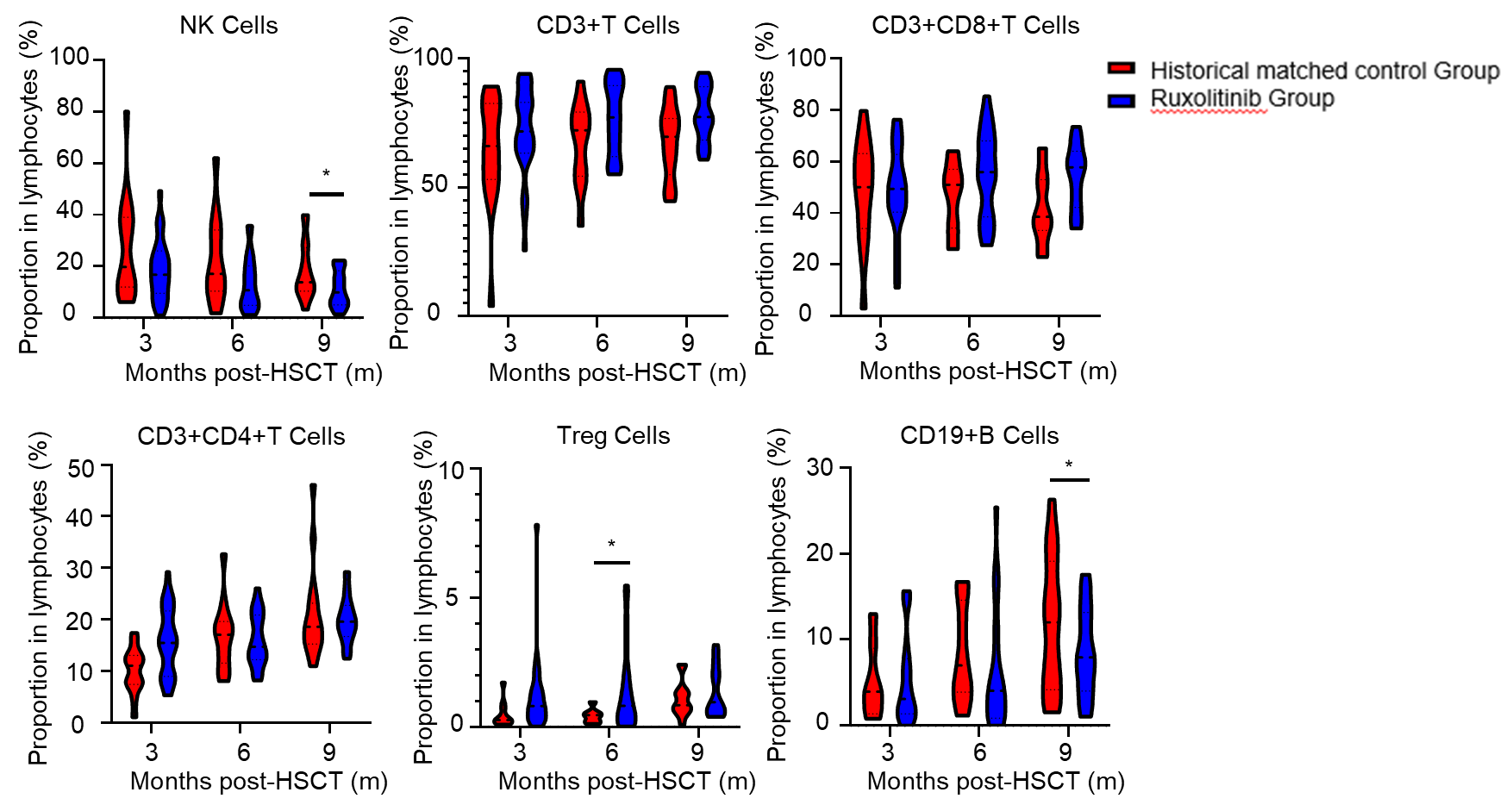

Supplement: Supplementary file 1 — Supplementary Data [file 41409_2024_2266_MOESM1_ESM.docx]
